# Novel Insight into Non-Genetic Risk Factors of Graves’ Orbitopathy

**DOI:** 10.3390/ijerph192416941

**Published:** 2022-12-16

**Authors:** Katarzyna Zawadzka-Starczewska, Bartłomiej Stasiak, Katarzyna Wojciechowska-Durczyńska, Andrzej Lewiński, Magdalena Stasiak

**Affiliations:** 1 Department of Endocrinology and Metabolic Diseases, Polish Mother’s Memorial-Hospital Research Institute, 281/289 Rzgowska St., 93-338 Lodz, Poland; 2 Institute of Information Technology, Lodz University of Technology, 215 Wolczanska St., 90-924 Lodz, Poland; 3 Department of Endocrinology and Metabolic Diseases, Medical University of Lodz, 281/289 Rzgowska St., 93-338 Lodz, Poland

**Keywords:** Graves’ orbitopathy, Graves’ disease, autoimmunity, TSH receptor antibodies, risk factors, LDL cholesterol, creatinine, oxidative stress

## Abstract

An assessment of the risk of Graves’ orbitopathy (GO) is an important challenge in Graves’ disease (GD) management. The purpose of this study was to compare non-genetic parameters in GD patients with and without GO in order to find novel risk factors and to verify the factors already reported. A total number of 161 people, 70 with GO and 91 non-GO patients were included in this study. GO was confirmed to be associated with smoking, older age, higher TSH receptor antibodies (TRAb) and lower thyroglobulin antibody (TgAb) levels and hypercholesterolemia. We demonstrated the latter correlation even for only a mild increase in LDL cholesterol. Importantly, our study provides novel potential GO risk factors, including higher serum creatinine levels, higher MCV and lower PLT. If further confirmed, these new, simple and easily accessible potential GO markers may constitute valuable auxiliary markers in GO risk assessments. We additionally proved that in moderate to severe GO, gender-related differences attenuate. No impact of vitamin D deficiency in GO development in patients with 25-hydroxyvitamin D [25(OH)D] > 20 ng/mL was found. The present report provides a set of GO risk factors, which can be used as a precise tool for an individual GO risk assessment.

## 1. Introduction

Graves’ disease (GD) is an autoimmune thyroid disorder (AITD), resulting from an uncontrolled production of antibodies against thyroid stimulating hormone (TSH) receptors (TRAb). These antibodies usually stimulate TSH receptor, leading to excessive thyroid hormone production [1]. A prevalence of GD is approximately 1% to 1.5%, with an incidence ranging from 20 to 30 new cases/100,000/year [2]. TRAb may exhibit a high affinity not only to the thyroid gland but also to other tissues, including eyes (orbitopathy), skin (dermatopathy) and bone and muscles (acropachy) [2,3]. Graves’ orbitopathy (GO), which is the most important extrathyroidal manifestation of GD, presents with lid retraction, exophthalmos, soft tissue involvement of the eye, spontaneous retrobulbar pain and pain on an attempted upward or downward gaze [4]. GO results in significantly reduced quality of life (QoL) and severe complications, including sight threatening conditions, such as optic neuropathy and/or corneal breakdown [4]. Our research team previously demonstrated that both genetic and non-genetic factors influence the clinical course of thyroid disorders [5]. Genetic susceptibility to GD was proved to be human leukocyte antigens (HLA)-related, with HLA-B*08:01, -B*39:06, -B*37:01, -C*07:01, -C*14:02, -C*03:02, -C*17:01, -DRB1*03:01, -DRB1*11:01, -DRB1*13:03, -DRB1*01:03, -DRB1*14:01, -DQB1*03:01, DQB1*02:01 being GD high risk alleles [6]. Recently, we have demonstrated that the risk of GO development is also HLA-dependent [7].Knowledge on genetic the background provides new tools for personalized patient management in GO prevention and treatment. However, it is a non-genetic factor that triggers GO in patients with a genetic predisposition. Therefore, the necessity to broaden the knowledge on factors, which induce GO or worsen its course is still growing, so as to further personalize the management. Several non-genetic factors are already known as GO-related, including smoking, higher TRAb levels, radioiodine therapy, uncontrolled hyper- or hypothyroidism and recently demonstrated higher total cholesterol (TC) and low density lipoprotein (LDL) cholesterol levels [4,8]. However, many other parameters, such as age, stress or vitamin D deficiency were postulated to be GO risk factors [9] but the results of different studies are inconsistent. Some laboratory parameters have never been compared between GO and non-GO GD patients in size-matched groups.

Knowledge on the risk factors associated with the onset or severity of GO can be fundamental in the prevention and treatment of GO. Thus, the aim of the present study was to compare clinical and laboratory features of patients belonging to GO and non-GO GD groups in order to find novel non-genetic GO risk factors and to verify the factors already reported. This analysis aims to provide novel tools for GO prevention and for the further development of a personalized GO management.

## 2. Materials and Methods

### 2.1. GD Rroup and Control Group

A total number of 161 people, 70 with GO and 91 non-GO patients who were diagnosed with GD in the Department of Endocrinology and Metabolic Diseases Polish Mother’s Memorial Hospital Research Institute, Lodz, Poland and the Department-associated outpatient clinic were included in this study. 

### 2.2. Inclusion Criteria

In all the patients included in this study, GD was diagnosed using the standard criteria [1], including hyperthyroidism, elevated TRAb level and typical ultrasound (US) patterns. GO was diagnosed on the basis of the presence of lid retraction, soft tissue involvement of the eye (redness, swelling) and exophthalmos, according to the guidelines [4]. Patients with GD and without GO were included in the prospective arm of this study in which long term follow up (>3 years) were applied to confirm patients’ group affiliation. All patients in whom GO symptoms occurred during the follow up were reclassified and included in the GO group. Patients with other acute or chronic diseases, which may have influenced laboratory results were excluded from the study.

### 2.3. Laboratory Procedures

Blood samples from all the patients were collected at the time of diagnosis. The procedure was performed by a single venipuncture, using the vacutainer system technique. Samples were collected in the morning (6.00–8.00 A.M.) after overnight fasting. Serum levels of TSH, free triiodothyronine (FT3), free thyroxine (FT4), thyroglobulin antibodies (TgAb), thyroperoxidase antibodies (TPOAb), TRAb, morning cortisol and 25-hydroxycholecalciferol were measured by the electrochemiluminescence immunoassay (ECLIA) with a Cobas e601 analyzer (Roche Diagnostics, Indianapolis, IN, USA). The erythrocyte sedimentation rate (ESR) was determined by Ves-Matic Cube 30 (Diesse, Monteriggioni, Italy). C-reactive protein (CRP), total cholesterol, LDL and HDL cholesterol, triglycerides, urea, creatinine, glucose, alanine aminotransferase (ALT), aspartate aminotransferase (AST) and bilirubin levels were determined by the VITROS^®^ 4600 Chemistry System (Ortho Clinical Diagnostics, Raritan, NJ, USA).

### 2.4. Medical History Analysis

Medical history data regarding radioiodine treatment or thyroidectomy were recorded on the basis of patients’ medical documentation. Active smokers at the time of GD diagnosis were recorded as smokers. Stressful events were evaluated on the basis of the Holmes–Rahe stress scale questionnaire with a result >150 considered positive for stressful event [10,11].

### 2.5. Statistical Analysis

Descriptive statistics of the collected data included median, mean, standard deviation and interquartile ranges. For comparisons between the groups, we used Student’s *t*-test for normally distributed variables (i.e., age, urea and total cholesterol), the Mann–Whitney U test for the other real-valued data and the chi-square test for categorical variables. The normality of data distributions was assessed by the Shapiro–Wilk test. Additionally, a correlation analysis, including Pearson’s coefficient and Spearman’s rank correlation coefficient calculations between the CAS score and TRAb (both in linear and logarithmic scale) was performed. For all the tests a *p* value < 0.05 was considered significant. The sample size was calculated on the basis of the region population size and GD prevalence in the Polish population with 95% confidence interval. Python statistical libraries (SciPy stats) were used for all the computations.

### 2.6. Ethics Procedures

Informed consent for all the performed procedures was obtained from all of the patients after a full explanation of the purpose and nature of all procedures used. The study was approved by the Ethics Committee of the Polish Mother’s Memorial Hospital-Research Institute, Lodz, Poland (approval code—108/2018).

## 3. Results

The mean age of the patients at the time of GD diagnosis was 41.3 ± 17.39 years, with a male to female ratio of 1: 4.57. The diagnosis of GD was based on laboratory results and the US pattern. For the whole GD group, median/IQR of TSH, FT4, FT3 and TRAb were 0.005/0.015 µIU/mL, 2.48/2.68 ng/dL, 7.95/13.43 pg/mL and 10.29/16.16 IU/L, respectively.

### 3.1. Comparison of GO and Non-GO Groups with Respect to Patients’ Age and Laboratory Results

A statistically significant difference was found for the mean age at GD diagnosis with patients in the GO group being older than those in the non-GO group (44.25 ± 14.61 vs. 39.00 ± 19.04 years) (Table 1). The mean time lag between a GD diagnosis and the onset of GO in the GO group was 32 months.

Severity of thyrotoxicosis was significantly higher in the non-GO group than in the GO group, with a higher TSH mean and lower mean concentration of FT3 and FT4 in the GO group than in non-GO group (0.18 ± 0.59 vs. 0.10 ± 0.48 µIU/mL, 9.59 ± 8.89 vs. 12.85 ± 8.06 pg/mL and 2.64 ± 2.09 vs. 3.58 ± 1.96 ng/dL, respectively). In the GO group, the mean TRAb concentration was significantly higher than in the non-GO group, while thyroglobulin antibody (TgAb) levels were significantly lower in the GO group than in the non-GO group (347.22 ± 894.99 vs. 406.41 ± 640.99) (Table 1). A statistically significant difference was found for serum glucose concentration with higher levels in the non-GO group than in the GO group (98.26 ± 18.74 vs. 94.10 ± 24.76 mg/dL). Creatinine levels were significantly higher in the GO group than in the non-GO group (0.68 ± 0.20 vs. 0.53 ± 0.16 mg/dL). Among the blood count parameters, the mean corpuscular volume (MCV) was significantly higher in the GO group than in the non-GO group (86.21 ± 6.35 vs. 82.93 ± 5.27 fl), while platelet (PLT) levels were lower in the GO group than in the non-GO group (238,370 ± 59,110 vs. 270,110 ± 72,740/µL). Among the lipid parameters, both TC and LDL cholesterol levels were significantly higher in the GO group than in the non-GO group (192.84 ± 53.39 vs. 161.96 ± 48.21 mg/dL and 117.28 ± 46.53 vs. 94.13 ± 39.74 mg/dL, respectively) (Table 1).

No statistically significant differences were found for other laboratory parameters (Table 1).

### 3.2. Comparison of GO and Non-GO Groups with Respect to Gender, Environmental Risk Factors and Treatment

Smoking was significantly more common in the GO group than in the non-GO group. Thyroidectomy was also performed more often in the GO group. No significant differences were found with respect to gender, stress or radioiodine treatment (Table 2).

### 3.3. Correlation Analysis between TRAb and CAS Score

The analysis of correlation between the TRAb and CAS scores revealed a weak, although definitely positive, correlation (r = 0.201, *p* = 0.161). The logarithm of TRAb showed a slightly higher correlation with CAS (r = 0.213, *p* = 0.137), while Spearman’s rank correlation coefficient was 0.245, *p* = 0.085.

## 4. Discussion

The risk factors of GO are not entirely clear and include genetic predispositions and interactions between endogenous and environmental factors [12]. Although genetic susceptibility seems to play an important role, GO is triggered and modulated by non-genetic factors. In the present study, we compared both clinical and environmental factors of GO groups and non-GO groups in order to find potential, novel and non-genetic GO risk factors as well as to analyze factors, which were previously postulated to be GO-associated.

Smoking and high TRAb levels were postulated as risk factors in many papers, including the current EUGOGO guidelines [4,8]. Our results confirmed a strong relationship between smoking and GO. Smoking is a strong oxidative stress inducer. Reactive oxygen species (ROS) were demonstrated to play a role in GO development. They stimulate orbital fibroblast proliferation, synthesis of glycosaminoglycans and inflammatory mediators [13]. Increased concentrations of ROS were found not only in blood, but also in the urine of GO patients [13]. The beneficial effect of selenium supplementation in GO is related to its antioxidant properties [13,14]. Tsai et al. also demonstrated the presence of oxidative DNA damage markers (higher level of 8-OHdG–8-hydroxy-2′-deoxyguanosine) in the urine of GO patients and a positive correlation between oxidative DNA damage and the clinical activity of GO [15].

TRAb levels were also, as previously demonstrated [16,17], significantly higher in GO patients. Since TSHR overexpression was confirmed in GO orbital tissues [18,19], TRAb levels are considered as an independent GO risk factor and a GO biomarker [8]. Many studies demonstrated higher levels of TRAb in GO than in non-GO GD patients. Wiersinga et al., in a prospective observational study on patients with newly diagnosed GD, postulated TRAb as an independent baseline determinant for the development of GO [20]. Diana et al. observed that TRAB levels correlate well with clinical activity and severity of GO [21]. Nicoli et al. showed a statistically significant, direct correlation between serum TRAb levels and CAS in patients with recent onset and untreated GO [16]; our results also revealed a positive correlation; however, it had not reached the level of statistical significance. Lantz et al. showed an increased risk of GO in patients with TRAb > 6.3 IU/L at the time of GD diagnosis [22]. In our cohort, the difference was significant but the mean levels of TRAb were much higher than 6.3 IU/L in both groups (i.e., 17.59 vs. 13.65 IU/L in GO and non-GO groups, respectively). Therefore, the actual threshold of TRAb for the risk of GO development should be further evaluated as it may be much higher than previously postulated [22].

There is a strong correlation between smoking and TRAb levels. Roos et al. demonstrated that during treatment TRAb levels normalize faster in non-smokers than in smokers [23]. Several authors reported that GO patients had higher TRAb levels, were statistically older and tended to smoke more frequently with the duration of hyperthyroidism being shorter when compared to patients without GO [24,25]. Our study confirmed these observations, as our GO group were older, had higher TRAb levels with smoking being significantly more common among them.

Unstable hyperthyroidism was also demonstrated as a GO risk factor [4] and the association between GO risk and the level of stimulatory TRAb was proved to be the most important. In our cohort, most patients in both GO and non-GO groups had unstable hyperthyroidism at the time of diagnosis, so we did not find such a correlation. Moreover, we observed a significantly less severe thyrotoxicosis in the GO group when compared to the non-GO group. A similar finding of lower FT4 levels in GO patients was previously reported by Goh et al. [26]. It is well known that GD-related biochemical thyrotoxicosis is usually much more severe in younger patients. As stated above, our study confirmed previous observations [27,28] that an older age at the onset of the disease is a GO risk factor. GO patients were significantly older than non-GO patients, and as GO occurred in some patients even after a two year onset of GD, we monitored our non-GO group during the >3-year follow up period in order to confirm that they were properly included in the non-GO group. Taking into account the above described findings, we supposed that the lower severity of thyrotoxicosis in the GO group may result from the older age of GO patients.

Thyroid antibody profiles in GO is a very interesting issue and it has been a subject in many studies. Expression of not only TSHR but also thyroglobulin (Tg) mRNA were found in GO orbital fibroblasts [29,30]. These reports suggested that Tg fibrocyte expression is a result of a substantial Tg promoter activity [29,30]. Although Khamisi et al. reported higher Tg levels in GO patients when compared to non-GO patients, they found TgAb levels to be lower in the GO group [31]. We have not analyzed Tg levels but TgAb concentrations were significantly lower in our GO patients. However, it should be underlined that the mean TgAb as well as TPOAb levels were elevated significantly above the reference range in both GO and non-GO groups. Similarly, other authors reported lower TgAb as well as TPO Ab in GO patients [26]. We also observed lower TPOAb levels but the difference did not reach a statistical significance.

Interestingly, we observed that serum glucose concentration was higher at the time of GD diagnosis in the non-GO group when compared with GO patients, which may be associated with a higher severity of thyrotoxicosis (higher FT3 and FT4 concentrations) in this group.

In addition, our study indicated significantly higher creatinine serum concentrations in the GO group than in non-GO group. We might postulate that this observation resulted mainly from the older age of the patients [32]. It is a plausible hypothesis but taking into account that urea levels did not significantly differ between GO and non-GO groups, and the mean and median urea values were actually lower in the GO group (although the difference were not statistically significant), this hypothesis seems questionable. Therefore, as higher creatinine levels in older age is most commonly associated with prerenal factors and increased urea levels, age does not seem to be the main cause of our finding of higher creatinine in GO patients. It should be underlined that we did not include patients with renal insufficiency, as their laboratory results would have been related mainly to renal disease. Therefore, our observation may indicate that higher (but still within the reference range) creatinine levels may constitute a novel GO risk factor. Obviously, further studies are necessary to confirm this observation and to find out whether there is any cut-off creatinine value, which can be considered as GO-related.

Moreover, significantly higher MCV in GO patients (when compared to the non-GO group) was demonstrated in our current study. Thyrotoxicosis was previously found to adversely affect hematopoiesis [33,34]. Krygier et al. demonstrated that thyrotoxic patients had lower but still normal MCV, which increased during treatment. They postulated that this phenomenon is inversely correlated with hepcidin levels [35]. Similarly, we observed lower MCV in our non-GO patients in whom thyrotoxicosis was more severe. However, oxidative stress may also play a role in this phenomenon as several authors observed that oxidative stress is associated with higher MCV levels [36]. Unfortunately, neither creatinine nor MCV levels had previously been analyzed as potential GO risk factors; therefore, our findings require further confirmation.

Our current research indicates significantly lower PLT levels in GO patients than in the group without GO. The autoimmune etiology of thrombocytopenia associated with Graves’ disease and the overlapping of the thyroid and platelets autoimmunity was well explained by Cordiano et al. [34]. Gill et al. showed that treatment of Graves’ disease resulted in a significant improvement in thrombocytopenia that was refractory to intravenous immunoglobulin and steroid treatment [37]. Thus, a significantly higher serum TRAb concentration and greater severity of the autoimmune process when compared to the group without GO may be a possible explanation for a significantly lower platelet concentration in GO patients.

In recent years, high serum total cholesterol (TC) and LDL cholesterol levels were demonstrated as an independent risk factors of GO [4,38,39]. Additionally, TC and LDL cholesterol were positively correlated with the GO CAS result [24,38]. No relationship between GO and high-density lipoprotein cholesterol (HDL) or triglycerides (TG) was observed [39]. In our cohort, we also observed higher TC and LDL concentrations in GO patients, with no differences in HDL and TG levels between GO and non-GO groups. Interestingly, we confirmed that there are significant differences in TC and LDL levels between GO and non-GO groups, even though in our groups mean and median TC values were within the reference range, and LDL mean and median values were only slightly above the upper reference value in the GO group. This finding underlines the significance of TC and LDL in GO development even in only mild LDL elevation. It was also demonstrated that cholesterol-lowering treatment improved the outcome in GO patients treated with intravenous glucocorticoid therapy (ivGC) [40,41]. Therefore, current guidelines recommend cholesterol lowering treatment in all GD patients [4]. Our present findings confirmed that even mild LDL elevation, or possibly even high normal LDL levels, are risk factors of GO development. Therefore, our study provides a further strong indication for cholesterol lowering therapy in GD in all patients even with a slight LDL elevation.

Recently, Heisel et al. postulated that serum 25-hydroxyvitamin D (25(OH)D) deficiency was an independent risk factor of GO [9]. The sizes of analyzed groups were similar to ours, but we have not observed such correlation. Interestingly, mean 25(OH)D concentrations were close to the lower normal limit in both the analyzed groups and in both studies. However, Heisel et al. observed lower levels in the GO group than in the non-GO group (24.8 vs. 29.4 ng/mL, *p* = 0.006) [8] while we observed even higher levels in the GO group (27 vs. 25 ng/mL) but the difference was not statistically significant. Since both studies did not reveal severe 25(OH)D deficiency in either of the analyzed groups, we believe that the differences within levels above 20 ng/mL are not associated with GO risk. The potential impact of severe vitamin deficiency (<10 ng/mL) on the risk of GO development requires further studies.

Topcu et al. showed a significantly higher number of negative stressful life events (SLEs) in GD patients when compared to the control group [42]. Kahaly et al., observed that six months prior to GO onset, 74 of 102 patients experienced a mean of 4 (range 0–13) SLEs [43]. In addition, patients with optic neuropathy had more stressful events than those without nerve involvement [43]. Additionally, the rates of anxiety and depression in GO were significantly higher than in healthy people [43]. However, it is unclear whether the anxiety and depression preceded GO, and more evidence is needed to understand the correlation between anxiety, depression and GO [44]. In our current study stressful events were common in both GO and non-GO groups and occurred in 70% and 63%, respectively. Although stressful events were more frequent in the GO group, the difference did not reach a statistical significance.

Although GD is more common in women, previous studies on the association between GO and gender provided inconsistent results. Many studies postulated a higher incidence in women when compared to men [45,46]. However, gender-related differences reduce in severe GO patients, and the patients with severe GO were more frequently men than women [47]. As our study was performed in a tertiary center, where patients mostly with moderate to severe GO are referred, we did not observe any correlation between GO risk and gender. In our center, we strictly follow the actual recommendations for GO prevention and radioiodine therapy is not applied in GD patients with a presence of any GO risk factor. This fact is the most probable explanation of the finding that we did not report any differences between our GO and non-GO groups with respect to the frequency of radioiodine therapy. Similarly, avoiding radioiodine treatment in patients at risk of GO resulted in a significantly higher frequency of thyroidectomies in the GO group than in the non-GO group.

The presented research has its strengths and limitations. It should be noted that our study included a selected group of patients who were referred to the tertiary center and, therefore, some group selection bias was possible. The statistical analysis was limited to a binary GO/non-GO scenario searching for significant differences between the groups, while correlation analysis, including CAS scores would probably reveal some more potentially interesting details. This direction will be considered in our future work. The strengths of the present study, on the other hand, lies in the strict inclusion criteria, long term follow-up of the non-GO group, a large number of analyzed parameters and unified laboratory assays for all the investigated patients.

## 5. Conclusions

Our present study confirmed that smoking, older age, higher TRAb and lower TgAb levels and higher TC and LDL concentrations are risk factors of GO development in patients with GD. We also further proved that in moderate to severe GO patients, gender-related differences attenuate. Moreover, our study suggests the existence of novel potential GO risk factors, including higher serum creatinine levels, higher MCV and lower PLT. These new, simple and easily accessible parameters may constitute valuable auxiliary markers in a GO risk assessment; however, their clinical significance for a GD/GO course requires further studies. We did not find any influence of vitamin deficiency on GO development in patients with 25(OH)D > 20 ng/mL. The present findings provides a set of GO risk factors, which can be used as a precise tool for an individual GO risk assessment, which can improve a personalized GO prevention and management.

## Figures and Tables

**Table 1:** Comparison of GO and non-GO groups in regard to patients’ age and laboratory results.

Parameter (Reference Range and Units)	GO		Non-GO		*p*-Value
	Mean ± SD (N)	Median/IQR	Mean ± SD (N)	Median/IQR	
Age at GD diagnosis (years)	44.25 ± 14.61 (71)	47.00/21.50	39.00 ± 19.04 (91)	40.00/28.00	0.049 *
TSH (0.27–4.2 µIU/mL)	0.18 ± 0.59 (59)	0.01/0.04	0.10 ± 0.48 (83)	0.01/0.01	0.049 *
FT3 (2.0–4.4 pg/mL)	9.59 ± 8.89 (56)	4.85/10.55	12.85 ± 8.06 (79)	11.63/11.95	0.003 *
FT4 (0.93–1.7 ng/dL)	2.64 ± 2.09 (57)	1.80/1.86	3.58 ± 1.96 (82)	3.15/2.86	<0.001 *
TPOAb (<34 IU/mL)	182.57 ± 195.05 (58)	77.50/268.85	211.54 ± 204.57 (71)	170.40/236.50	0.23
TgAb (<115 IU/mL)	347.22 ± 894.99 (58)	25.45/218.22	406.41 ± 640.99 (67)	250.00/484.30	<0.001 *
TRAb (<1.75 IU/L)	17.59 ± 13.79 (68)	13.72/26.95	13.65 ± 13.52 (88)	9.20/13.25	0.021 *
Morning cortisol (>10 µg/dL)	12.87 ± 5.08 (15)	11.00/5.27	14.12 ± 6.80 (18)	14.47/8.38	0.56
ESR (<12 mm/h)	21.40 ± 20.18 (40)	17.00/23.50	19.00 ± 20.38 (30)	15.50/20.00	0.34
CRP (<1.00 mg/dL)	0.71 ± 0.56 (54)	0.50/0.10	0.89 ± 1.10 (48)	0.50/0.13	0.86
Glucose (60–99 mg/dL)	94.10 ± 24.76 (59)	89.00/11.50	98.26 ± 18.74 (52)	96.00/11.00	0.006 *
Urea (19.3–42.3 mg/dL)	31.56 ± 10.25 (48)	31.00/11.00	32.40 ± 7.49 (45)	32.00/10.00	0.65
Creatinine (0.66–1.25 mg/dL)	0.68 ± 0.20 (58)	0.66/0.23	0.53 ± 0.16 (54)	0.50/0.21	<0.001 *
ALT (women < 35 U/L, men < 50 U/L)	30.22 ± 16.90 (58)	24.00/17.50	32.27 ± 16.78 (61)	29.00/22.00	0.45
AST (women < 36 U/L, men < 59 U/L)	26.71 ± 8.51 (59)	25.00/9.00	29.59 ± 10.18 (60)	27.00/13.25	0.11
Bilirubin (0.2–1.3 mg/dL)	0.62 ± 0.19 (40)	0.65/0.22	0.66 ± 0.43 (46)	0.57/0.39	0.66
WBC (4–10 × 10^3^/µL)	6.96 ± 2.21 (63)	6.49/2.87	6.71 ± 2.37 (64)	6.62/3.02	0.57
RBC (women 3.8–5.8 × 10^6^/µL, men 4.5–6.5 × 10^6^/µL)	4.58 ± 0.51 (62)	4.49/0.62	4.68 ± 0.50 (65)	4.60/0.65	0.23
HGB (women 12–15, men 13–18 g/dL)	13.39 ± 1.59 (62)	13.30/1.77	13.09 ± 2.00 (65)	13.00/1.70	0.21
HCT (women 36–45, men 40–54%)	39.62 ± 4.44 (62)	39.00/4.97	38.58 ± 3.46 (65)	38.70/3.80	0.25
PLT (150–400 × 10^3^/µL)	238.37 ± 59.11 (62)	235.00/79.25	270.11 ± 72.74 (65)	259.00/89.00	0.02 *
MCV (women 78–93 fl, men 82–94 fl)	86.21 ± 6.35 (58)	86.95/7.25	82.93 ± 5.27 (60)	82.55/7.67	<0.001 *
MCHC (32–37 g/dL)	33.78 ± 1.08 (62)	34.00/1.10	33.73 ± 1.01 (65)	33.70/1.00	0.39
TC Total cholesterol (<200 mg/dL)	192.84 ± 53.39 (64)	189.00/82.75	161.96 ± 48.21 (54)	152.00/70.25	0.001 *
LDL cholesterol (<100 mg/dL)	117.28 ± 46.53 (64)	103.50/86.00	94.13 ± 39.74 (54)	85.00/31.75	0.006 *
HDL cholesterol (>40 mg/dL)	51.80 ± 15.84 (64)	50.00/21.00	47.94 ± 15.79 (53)	46.00/19.00	0.13
triglycerides (<150 mg/dL)	121.53 ± 54.28 (64)	111.50/77.50	106.93 ± 47.41 (54)	91.50/56.75	0.13
25(OH)D (>30 ng/mL)	27.05 ± 10.37 (51)	25.30/15.80	25.24 ± 15.21 (45)	22.60/9.40	0.12

* The differnce is statistically significant, with *p* < 0.05.

**Table 2:** Comparison of GO and non-GO groups in regard to gender, environmental risk factors and treatment.

Parameter	GO	Non-GO	*p*-Value
Male/Female ratio	14/57	15/76	0.59
Smoking (Yes/No)	32/37	13/71	<0.001 *
Stressful events (Yes/No)	46/20	46/27	0.27
^131^I treatment (Yes/No)	21/50	17/72	0.12
Thyroidectomy (Yes/No)	16/55	2/87	<0.001 *

* The differnce is statistically significant, with *p* < 0.05.

## Data Availability

The source data are available on reasonable request from the corresponding author.

## Abbreviations

AITD	autoimmune thyroid diseases
ALT	alanine aminotransferase
AST	aspartate aminotransferase
CAS	clinical activity score
CRP	C-reactive protein
ESR	erythrocyte sedimentation rate
EUGOGO	European Group of Graves’ Ophthalmopathy
FT3	free triiodothyronine
FT4	free thyroxine
GD	Graves’ disease
GO	Graves’ orbitopathy
HCT	hematocrit
HDL	high-density lipoprotein
HGB	hemoglobin
HLA	human leukocyte antigens
LDL	low-density lipoprotein
mRNA	messenger RNA
MCHC	mean corpuscular hemoglobin concentration
MCV	mean corpuscular volume
PLT	platelet count
QoL	quality of life
RAI	radioactive iodine
ROS	reactive oxygen species
SLEs	stressful life events
TC	total cholesterol
TG	triglycerides
Tg	thyroglobulin
TgAb	thyroglobulin antibodies
TPOAb	thyroid peroxidase antibodies
TRAb	TSH-receptor antibodies
TSH	thyroid stimulating hormone (thyrotropin)
US	ultrasound
8-OHdG	8-hydroxy-2′-deoxyguanosine
25(OH)D	25-hydroxyvitamin D
